# Novel role of AMPK in cocaine reinforcement via regulating CRTC1

**DOI:** 10.1038/s41398-022-02299-w

**Published:** 2022-12-31

**Authors:** Xiao-Xing Liu, Fang-Lin Liu, Xin Li, Tang-Sheng Lu, Yi-Xiao Luo, Min Jian, Kai Yuan, Shi-Qiu Meng, Yan-Ping Bao, Jie Shi, Lin Lu, Ying Han

**Affiliations:** 1 Peking University Sixth Hospital, Peking University Institute of Mental Health, NHC Key Laboratory of Mental Health (Peking University), National Clinical Research Center for Mental Disorders (Peking University Sixth Hospital), Beijing, 100191 China; 2 National Institute on Drug Dependence and Beijing Key Laboratory of Drug Dependence Research, Peking University, Beijing, 100191 China; 3 Department of Neurobiology, School of Basic Medical Sciences, Peking University Health Science Center, Beijing, 100191, China; 4 Key Laboratory of Molecular Epidemiology of Hunan Province, School of Medicine, Hunan Normal University, Changsha, 410081 China; 5 School of Public Health, Peking University, Beijing, 100871 China; 6 Peking-Tsinghua Center for Life Sciences and PKU-IDG/McGovern Institute for Brain Research, Peking University, Beijing, 100871 China

**Keywords:** Addiction, Molecular neuroscience

## Abstract

Repeated cocaine exposure causes compensatory neuroadaptations in neurons in the nucleus accumbens (NAc), a region that mediates reinforcing effects of drugs. Previous studies suggested a role for adenosine monophosphate-activated protein kinase (AMPK), a cellular energy sensor, in modulating neuronal morphology and membrane excitability. However, the potential involvement of AMPK in cocaine use disorder is still unclear. The present study employed a cocaine self-administration model in rats to investigate the effect of AMPK and its target cyclic adenosine monophosphate response element binding protein-regulated transcriptional co-activator 1 (CRTC1) on cocaine reinforcement and the motivation for cocaine. We found that intravenous cocaine self-administration significantly decreased AMPK activity in the NAc shell (NAcsh), which persisted for at least 7 days of withdrawal. Cocaine reinforcement, reflected by self-administration behavior, was significantly prevented or enhanced by augmenting or suppressing AMPK activity pharmacologically and genetically, respectively. No difference in sucrose self-administration behavior was found after the same manipulations. The inhibition of AMPK activity in the NAcsh also increased the motivation for cocaine in progressive-ratio schedules of reinforcement, whereas the activation of AMPK had no effect. The knockdown of CRTC1 in the NAcsh significantly impaired cocaine reinforcement, which was rescued by pharmacologically increasing AMPK activity. Altogether, these results indicate that AMPK in the NAcsh is critical for cocaine reinforcement, possibly via the regulation of CRTC1 signaling. These findings may help reveal potential therapeutic targets and have important implications for the treatment of cocaine use disorder and relapse.

## Introduction

Substance use disorder is a chronic relapsing disorder. Many efforts have been devoted to elucidating the mechanisms and developing novel therapeutic strategies [1]. Previous studies showed that long-term behavioral abnormalities are corelated with abnormal neuroadaptations in mesocorticolimbic systems [2, 3]. Drug exposure also induces persistent structural changes in limbic regions, such as the nucleus accumbens (NAc), which is a hub for the reinforcing effects of drugs [4]. However, remaining unclear are the molecular mechanisms that underlie these structural changes in the NAc.

Adenosine monophosphate (AMP)-activated protein kinase (AMPK) is a heterotrimeric serine/threonine protein kinase that is highly expressed in the central nervous system. AMPK regulates cellular energy homeostasis, cell structure and polarity, and normal growth and development [5–7]. Studies showed that AMPK activation was sufficient to induce dendritic spine loss and regulate age-related synaptic remodeling [8–10], suggesting a critical role for AMPK under pathological conditions. Studies with *Drosophila* and rodents confirmed that AMPK is involved in neurodegeneration [11, 12]. Although few investigations have focused on the effects of modulating AMPK activity on addiction-related behaviors, our previous study showed that activating AMPK in the NAc core (NAcc) decreased the cue-induced reinstatement of cocaine-seeking behavior [13]. However, little is known about the effect of modulating AMPK activity in the NAc shell (NAcsh) on the development and persistence of addictive behaviors.

Previous evidence indicates that AMPK could regulate transcription through phosphorylation of many transcriptional regulators [14], among which cyclic adenosine monophosphate response element binding protein (CREB)-regulated transcriptional co-activators (CRTCs) are important [15]. Activated AMPK phosphorylates and inhibits CRTCs by binding them to 14-3-3 and cytoplasmic sequestration, thus decreasing CREB activity and blocking CREB-dependent transcription [15, 16]. In addition to transcriptional coactivation, the CRTC family is also involved in various biological functions, such as lifespan prolongation, learning and memory, and glucose metabolism [17]. Remaining unclear is the role of CRTCs in cocaine-related behaviors.

The present study examined the effect of AMPK-CRTC1 signaling on cocaine reinforcement. Our results showed that pharmacological and genetic activation of AMPK in the NAcsh significantly prevented cocaine self-administration. The inhibition of AMPK had an opposite effect, enhancing both cocaine reinforcement and the motivation for cocaine. The overexpression of CRTC1 rescued the decrease in cocaine reinforcement that was induced by AMPK activation. These findings provide evidence that AMPK activity in the NAcsh contributes to cocaine reinforcement by regulating the activity of CRTC1.

## Materials and methods

### Subjects

Male Sprague Dawley rats, weighing 240–260 g, were obtained from the Laboratory Animal Center, Peking University Health Science Center. They were housed five per cage in a temperature- (23 ± 2 °C) and humidity- (50 ± 5%) controlled animal facility with *ad libitum* access to food and water. They were kept on a reverse 12 h/12 h light/dark cycle. The behavioral experiments were conducted during the dark phase of the cycle. All of the experiments were performed according to the National Institutes of Health Guide for the Care and Use of Laboratory Animals and were approved by the Biomedical Ethics Committee of Peking University on animal use and protection.

### Design, construction, and validation of adenoviral vectors for AMPK subunits

Constitutively active (CA) AMPKα2 cDNA constructs (T172D mutant) and dominant-negative (DN) AMPKα2 cDNA constructs (K45R mutant) were designed and constructed according to previous studies [18, 19]. The CA-AMPK construct encodes residues 1–312 of AMPKα2 that are mutated on the threonine 172 residue to aspartic acid (T172D). DN-AMPK contains full-length AMPKα2 that is mutated on the lysine 45 residue to arginine (K45R). Successful mutagenesis was validated by sequencing. All AMPK cDNAs were subcloned into the pHBAd-CMV vector. All vectors contained the enhanced green fluorescence protein (eGFP) coding sequence.

### Design, construction, and validation of adeno-associated virus vectors for CRTC1

CA-CRTC1 constructs were created by introducing two point-mutations, S151A and S245A. The CRTC1 RNAi sequence for shCRTC1 was CGAACAATCCGCGGAAATTTA. For the overexpression experiment, the CAG promoter and GFP-T2A-CA-CRTC1 were inserted into an adeno-associated virus 9 (AAV9) vector. For the knockdown experiment, the H1 promoter and shCRTC1 sequence were inserted into an AAV9 vector. All vectors contained the eGFP coding sequence.

### Intracranial and intravenous surgery

Rats (weighing 280–300 g when surgery began) were anesthetized with sodium pentobarbital (50 mg/kg, i.p.). Catheters were inserted into the right jugular vein, with the tip terminating at the opening of the right atrium as described previously [20]. Guide cannulas (23 gauge; Plastics One, Roanoke, VA, USA) were bilaterally implanted 1 mm above the NAcsh. The cannula was placed at a 16° angle toward the midline to avoid penetration of the lateral ventricle. The coordinates [21] for the NAcsh were the following: anterior/posterior, +1.8 mm; medial/lateral, ±3.2 mm, dorsal/ventral, −6.6 mm [22, 23]. The cannulas were anchored to the skull with stainless-steel screws and dental cement. A stainless-steel stylet blocker was inserted into each cannula to keep it patent and prevent infection. The rats were allowed to recover for 7 days after surgery.

### Intracranial injections

The AMPK activator 5-amino-1-β-D-ribofuranosyl-imidazole-4-carboxamide (AICAR) was purchased from Toronto Research Chemicals. The AMPK inhibitor compound C was purchased from Sigma (St. Louis, MO, USA). Compound C was dissolved in vehicle solution that contained 80% sterile saline, 10% dimethylsulfoxide (DMSO), and 10% cremophore EL (Sigma-Aldrich). AICAR was dissolved in saline. All drugs were freshly prepared before delivery. The drug doses (5 μg/μl AICAR, 3 μg/μl compound C) were based on previous studies [24–26]. The infusion volume for all drugs was 0.5 μl. The drugs were infused bilaterally in the NAcsh using Hamilton syringes that were connected to 30 gauge injectors (Plastics One) that reached 1 mm below the guide cannula over 1 min. The injection needle was kept in place for an additional 1 min to allow drug diffusion.

The experimental procedure that was used for the virus injections was based on previous studies [26]. The rats were anesthetized with sodium pentobarbital. The adenoviruses (1 × 10^11^ pfu/ml) and AAVs (1 × 10^12^ pfu/ml) were delivered bilaterally over 10 min at an infusion rate of 0.05 μl/min (total volume, 0.5 μl per side) using Hamilton syringes that were connected to 30 gauge injectors (Plastics One) that reached 1 mm below the guide cannula. The injectors were left in place for an additional 5 min to allow diffusion before removing them.

### Cocaine self-administration procedures

The procedures for cocaine self-administration training were based on previous studies with minor modifications [20]. The chambers (AniLab Software and Instruments, Ningbo, China) were equipped with two nosepoke operandi (ENV-114M; Med Associates, St. Albans, VT, USA) that were 9 cm above the floor of the chambers. Nosepokes in one (active) operandum led to cocaine infusions that were accompanied by a 5 s tone-light cue. Nosepokes in the other (inactive) operandum were recorded but had no consequences. The rats were trained to self-administer intravenous cocaine hydrochloride (0.5 mg/kg/infusion) during three 1 h sessions daily that were separated by 5 min over 13–15 days. Each injection was accompanied by the illumination of a cue light above the active nosepoke, followed by an additional 12.5 s timeout period when the cue and house lights were extinguished and additional nosepoke responses had no programmed consequence. Each session began with the illumination of a houselight that remained on for the entire session. The fixed-ratio (FR) requirement was increased from FR1 to FR3, then to FR5, and training continued until cocaine intake stabilized. The number of cocaine infusions was limited to 30 per hour. At the end of the training phase, the groups in the different experimental conditions were matched for their cocaine intake during training. The rats then underwent a between-session dose-response test with one of five cocaine injection doses (1.0, 0.3, 0.1, 0.03, and 0 mg/kg, i.v.) that were presented each hour in descending dose order. Catheter patency was verified after testing by brief anesthesia with sodium methohexital (0.1 mg/0.1 ml).

Following the dose-response test, the rats self-administered the maintenance dose of cocaine in daily 3 h sessions until cocaine intake stabilized. After stabilization, the rats self-administered one of two doses of cocaine (0.25 or 0.75 mg/kg, i.v.). The response requirement for each successive injection increased progressively according to the following series: 1, 2, 4, 6, 9, 12, 15, 20, 25, 32, 40, 50, 62, etc., as described previously [27]. Each dose was tested in two consecutive daily sessions in a counterbalanced order. Progressive-ratio breakpoints were determined as the final ratio of responses/injection that were achieved before a 1-h period when no further injections were earned.

### Sucrose self-administration procedure

The experimental conditions were identical to those that were described above for cocaine self-administration, with the exception that active nosepoke responses led to the delivery of 0.1 ml of 10% sucrose into a liquid receptacle.

### Histology

After the behavioral experiments, the rats were anesthetized with sodium pentobarbital (100 mg/kg, i.p.) and perfused with 0.01 mol/L phosphate-buffered saline (PBS) and 4% paraformaldehyde (PFA; pH 7.4). The brains were then extracted and post-fixed in 4% PFA for 24 h. The brains were then cryoprotected in 30% sucrose, which was dissolved in 0.2 mol/L phosphate buffer. The brains were coronally sectioned at 20 μm using a sliding microtome. Brain slices were examined using an Olympus BX53 fluorescent microscope for eGFP expression in adenoviral vector-injected rats and the brain slices were additionally counterstained with DAPI in adeno-associated virus-injected rats. The rats with misplaced cannulae were excluded from the statistical analysis.

### Western blot

The Western blot procedures were based on our previous studies [28–30]. Equal amounts of protein (10–20 μg) for each samples were loaded into sodium dodecyl sulfate-polyacrylamide gel electrophoresis (SDS-PAGE; 10% acrylamide/0.27% *N*,*N’*-methylenebisacryalamide) for approximately 40 min at 80 V in stacking gel and approximately 1 h at 120 V in resolving gel. Proteins were electrophoretically transferred to Immobilon-P transfer membranes (Millipore, Bedford, MA, USA) at 250 mA for 2.5 h. Membranes were blocked with blocking buffer (5% bovine serum albumin [BSA] in TBST) for 2 h at room temperature. They were then incubated overnight at 4 °C with anti-phosphorylated-AMPK (p-AMPK) antibody (1:1000; Cell Signaling Technology, Danvers, MA, USA), anti-AMPK antibody (1:1000; Cell Signaling Technology, Danvers, MA, USA), or anti-β-actin antibody (1:1000; Santa Cruz Biotechnology, Santa Cruz, CA, USA) in TBST plus 5% bovine serum albumin (BSA). After three 5-min washes in TBST buffer, the blots were incubated for 45 min at room temperature on a shaker with the corresponding horseradish peroxidase-conjugated secondary antibody (goat anti-mice IgG for β-actin and goat anti-rabbit IgG for the others; 1:5000; Santa Cruz Biotechnology, Santa Cruz, CA, USA). The blots were washed three times for 5 min each in TBST, and immunostaining was visualized with a layer of Super Signal Enhanced chemiluminescence substrate (Detection Reagents 1 and 2, 1:1 ratio, Pierce Biotechnology, Rockford, IL, USA). The immunoblots were then screened using the ChemiDoc MP System (Bio-Rad, Hercules, CA, USA) for 5–60 s. Band intensities were quantified using Quantity One 4.4.0 software (Bio-Rad, Hercules, CA, USA).

### Statistical analysis

All of the statistical analyses were performed using SPSS 20.0 software (SPSS, Chicago, IL, USA). The rats were randomly allocated to different experimental groups. The sample sizes, and tests for each experiment can be found in the figure legends. The statistical analyses were performed using repeat-measures analysis of variance (ANOVA) for all of the experiments except the Western blot analysis (one-way ANOVA). *Post hoc* analyses of significant effects in the ANOVAs were performed using the Tukey test (for details, see the Results section). The data are expressed as mean ± SEM. Values of *p* < 0.05 were considered statistically significant.

## Results

### Cocaine self-administration regulates AMPK activity in the NAcsh

**Fig. 1:**
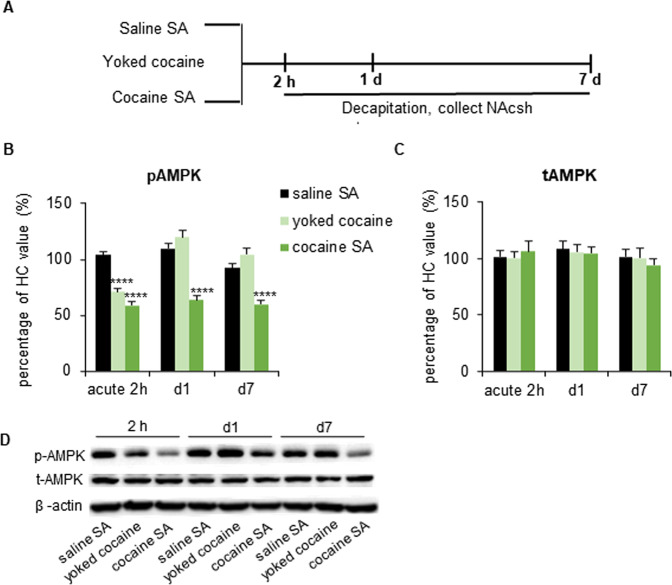
Time course of changes in AMPK activity in the NAcsh following intravenous cocaine self-administration (SA). **A** Timeline of the experiment. The rats were trained for saline self-administration, chronic yoked cocaine administration, or cocaine self-administration or maintained in their home cage (HC). They were then decapitated 2 h, 1 day, or 7 days later. **B**–**D**, Phosphorylated AMPK (**B**) and total AMPK (**C**) protein levels and representative Western blots (**D**) in the NAcsh 2 h, 1 day, or 7 days after cocaine administration. The data are expressed as a percentage of total AMPK and phosphorylated AMPK in home cage rats (*n* = 7/group). *Post hoc* analyses were performed using the Tukey test. ****p < 0.0001, compared with saline self-administration group.

To investigate dynamic changes in AMPK activity that are related to addictive behaviors, we employed a cocaine self-administration model in rats (Fig. 1A). The results showed a decrease in p-AMPK (*p* = 0.000; Fig. 1B, D) 2 h after the last cocaine training session. Interestingly, the lower expression levels persisted for at least 7 days of withdrawal in the NAcsh (*p* = 0.000). In chronic yoked rats that received the same number and temporal pattern of cocaine injections passively throughout training, p-AMPK levels in the NAcsh decreased only 2 h after the last cocaine injection (*p* = 0.000; Fig. 1B) and returned to baseline levels after 1 day of withdrawal (*p* = 0.346; Fig. 1B). No change in total AMPK levels was found in the NAcsh (Fig. 1C, D). These results demonstrated that passive and active cocaine self-administration differentially affected AMPK activity in the NAcsh, implying a possible regulatory role for AMPK in cocaine reinforcement behaviors.

### Pharmacological modulation of AMPK activity in the NAcsh regulates the behavioral response to cocaine

**Fig. 2:**
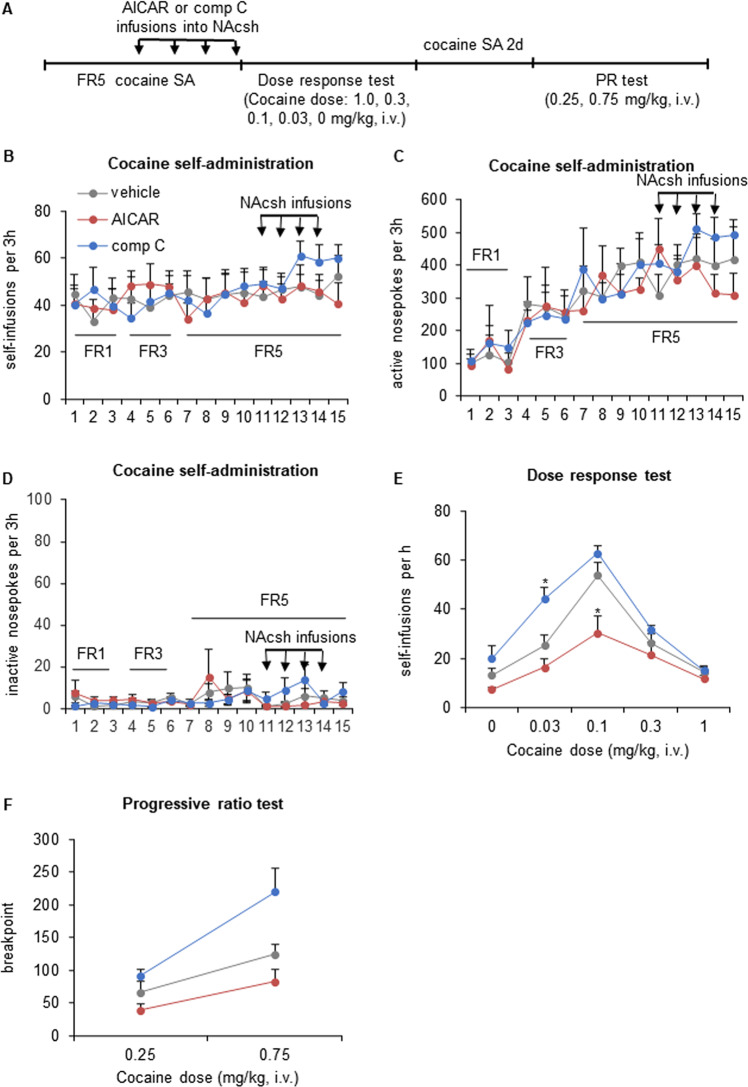
Pharmacological modulation of AMPK activity in the NAcsh controls cocaine reinforcement and motivation. **A** Timeline of the experiment. The rats were trained to self-administer intravenous injections of cocaine under an FR1 schedule in daily 3 h sessions. The response requirement was gradually increased to FR5, and training continued until cocaine intake stabilized. Following acquisition and stabilization, the rats received intra-NAcsh infusions of vehicle or the AMPK activator AICAR or AMPK inhibitor compound C (comp C). The rats then underwent a between-session dose-response test and progressive-ratio (PR) test. Infusions (**B**), active nosepokes (**C**), and inactive nosepokes (**D**) during daily 3 h self-administration sessions. **E** Repeated infusions of AICAR or comp C in the NAcsh produced opposite changes in cocaine self-administration dose-response curves after the cessation of treatment (*n* = 7–9/group). **F** Comp C-treated animals subsequently achieved a higher ratio of lever-press responses per cocaine injection before ceasing self-administration in the progressive-ratio test at the 0.75 mg/kg dose of cocaine (*n* = 7–8/group). The data are expressed as mean ± SEM. *Post hoc* analyses were performed using the Tukey test. *p < 0.05, compared with vehicle group.

The above results demonstrated dynamic changes in AMPK activity in the NAcsh after cocaine self-administration. We further investigated the consequences of regulating AMPK activity in the NAc on behavioral effects of cocaine. Rats were trained to self-administer cocaine (0.5 mg/kg/injection) in daily 3-h sessions until cocaine intake stabilized. After stabilization, the rats received infusions of the AMPK activator AICAR (2.5 μg per side), AMPK inhibitor compound C (1.5 μg per side), or vehicle in the NAcsh immediately before four consecutive sessions (Fig. 2A). Neither AICAR nor compound C infusions in the NAcsh significantly altered baseline cocaine self-administration during the treatment regimen (Fig. 2B–D).

The rats were then tested in a between-session dose-response procedure with one of five cocaine doses that were presented each hour in descending dose order. All treated rats exhibited typical inverted U-shaped dose-response curves that were dose-dependent (dose effect: *F*_4,84_ = 52.000, *p* = 0.000; Fig. 2E). A descending limb was observed, whereby higher injection doses prolonged the duration of cocaine reward, resulting in fewer self-injections over time than with lower injection doses. The repeated-measures ANOVA revealed significant effects of group (*F*_2,21_ = 13.564, *p* = 0.000) and a group × dose interaction (*F*_8,84_ = 3.814, *p* = 0.001). The prior intra-NAcsh infusion of AICAR decreased cocaine self-administration at a threshold dose of 0.1 mg/kg/injection at the peak of the dose-response curve compared with vehicle-infused controls (*p* = 0.012; Fig. 2E). The prior intra-NAcsh infusion of compound C increased cocaine self-administration at a threshold dose of 0.03 mg/kg/injection on the ascending limb of the dose-response curve compared with vehicle-infused controls (*p* = 0.013; Fig. 2E). The above results suggested that inhibition of AMPK activity in the NAcsh enhanced cocaine reinforcement and behavioral response to cocaine, whereas augmenting AMPK activity had an opposite effect. It should be noted that we did not include a group of rats that received no vehicle infusion so as to assess the possible influence of DMSO + Cremophore, so we cannot exclude an effect of this vehicle on the behaviors examined.

After the dose-response test, cocaine self-administration was re-stabilized at 0.5 mg/kg/injection. Subsequently, the motivation for cocaine was assessed using a progressive-ratio schedule of reinforcement to determine the highest ratio of nosepoke responses per cocaine injection that rats would achieve before they voluntarily ceased self-administration (i.e., the breakpoint). Breakpoints were measured at two cocaine injection doses (0.25 and 0.75 mg/kg/injection). The rats generally worked harder for the higher injection dose (dose effect: *F*_1,19_ = 46.070, *p* = 0.000; Fig. 2F), indicating greater motivation to self-administer the higher dose of cocaine. The repeated-measures ANOVA revealed a significant effect of group (*F*_2,19_ = 7.412, *p* = 0.004) and a group × dose interaction (*F*_2,19_ = 5.663, *p* = 0.012). Rats that received intra-NAcsh infusions of compound C achieved higher breakpoints at the 0.75 mg/kg dose of cocaine compared with vehicle-infused controls (*p* = 0.050), indicating greater motivation for cocaine. Conversely, rats that received intra-NAcsh AICAR infusions achieved lower breakpoints at both injection doses, but no significant difference from vehicle-infused controls was found.

### Modulation of AMPK activity in the NAcsh by adenovirus transfer regulates the behavioral response to cocaine

**Fig. 3:**
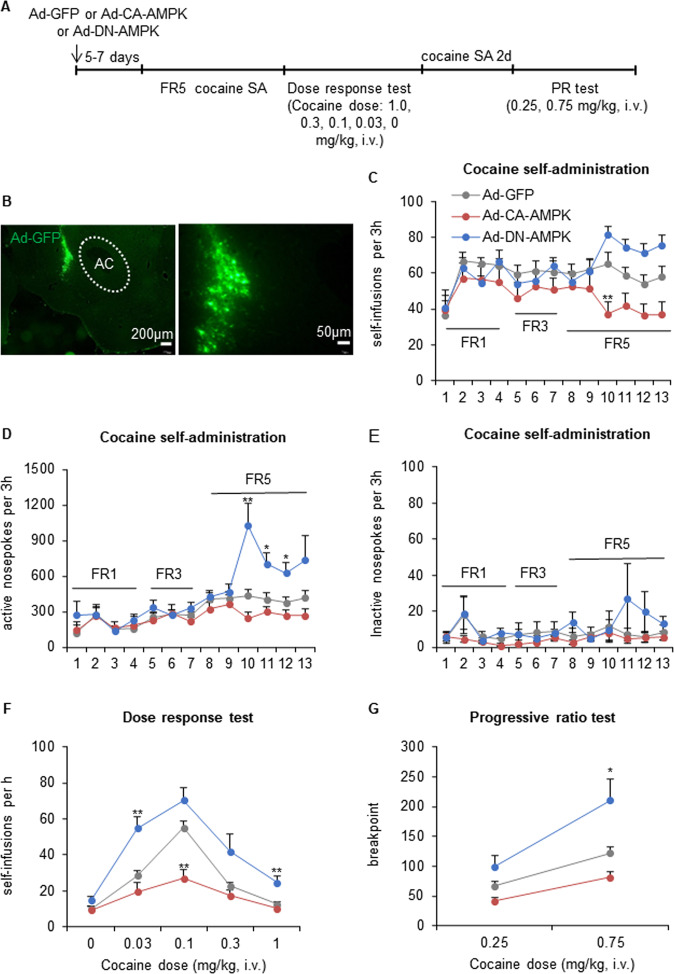
Modulation of AMPK activity by adenovirus gene transfer in the NAcsh controls cocaine reinforcement and motivation. **A** Timeline of the experiment. The rats received intra-NAcsh infusions of Ad-GFP, Ad-constitutively active (CA)-AMPK, or Ad-dominant negative (DN)-AMPK. The rats were then trained to self-administer intravenous injections of cocaine under an FR1 schedule in daily 3 h sessions. The response requirement was gradually increased to FR5, and training continued until cocaine intake stabilized. The rats then underwent a between-session dose-response test and progressive-ratio test. **B** Photomicrographs of Ad-GFP injection sites in the NAcsh (scale bar = 200 μm [left], 50 μm [right]). **C**–**E** Infusions (**C**), active nosepokes (**D**), and inactive nosepokes (**E**) during daily 3 h self-administration sessions. **F** Increasing or decreasing AMPK activity by adenovirus expressing CA-AMPK or DN-AMPK in the NAcsh produced opposite changes in cocaine self-administration dose-response curves (*n* = 9–11/group). **G** Animals that received adenovirus-expressing DN-AMPK subsequently achieved a higher ratio of lever-press responses per cocaine injection before ceasing self-administration in a progressive-ratio test at the 0.75 mg/kg dose of cocaine (*n* = 8–11/group). The data are expressed as mean ± SEM. *Post hoc* analyses were performed using the Tukey test. *p < 0.05, **p < 0.01, compared with Ad-GFP group.

Previous studies have shown that AICAR and compound C can function independently of AMPK [31–33]. Therefore, we further confirmed the effects of manipulating AMPK activity on cocaine reinforcement and motivation by expressing CA-AMPK (T172D mutation in the α2 subunit of AMPK) or DN-AMPK (K45R mutation in the α2 subunit of AMPK) in the NAcsh. We first examined the localization of adenovirus microinjections in the NAcsh (Fig. 3A, B, Supplementary Fig. 1A, B). The rats were given bilateral intra-NAcsh infusions of Ad-GFP, Ad-CA-AMPK, or Ad-DN-AMPK 5–7 days before the onset of cocaine self-administration. The repeated-measures ANOVA of self-infusions revealed a significant group × training session interaction (*F*_24,324_ = 3.435, *p* = 0.000). The repeated-measures ANOVA of active nosepokes revealed a significant effect of group (*F*_2,27_ = 5.905, *p* = 0.007) and a group × training session interaction (*F*_24,324_ = 3.990, *p* = 0.000). No significant differences in overall cocaine self-administration were found on the FR1 or FR3 schedule during acquisition training (Fig. 3C, D). On the FR5 schedule, Ad-CA-AMPK-infused rats exhibited lower cocaine infusions compared with Ad-GFP-infused controls (infusions on day 10: *p* = 0.009; Fig. 3C). Ad-DN-AMPK-infused rats responded much more vigorously for cocaine infusions (active nosepokes on day 10: *p* = 0.002, active nosepokes on day 11: *p* = 0.012, active nosepokes on day 12: *p* = 0.031; Fig. 3D). No group differences were found for responding on the inactive nosepoke operandum during cocaine self-administration training (*p* > 0.05; Fig. 3E). Thus, prolonged increases or decreases in AMPK activity in the NAcsh affected the acquisition of cocaine self-administration under conditions of higher motivation.

The rats were subsequently tested in a between-session dose-response procedure. All cocaine-treated rats exhibited inverted U-shaped dose-response curves that were dose-dependent (dose effect: *F*_4,108_ = 64.883, *p* = 0.000; Fig. 3F). The repeated-measures ANOVA revealed a significant effect of group (*F*_2,27_ = 16.234, *p* = 0.000) and a group × dose interaction (*F*_8,108_ = 6.244, *p* = 0.000). Ad-GFP-infused control rats exhibited typical inverted U-shaped dose-response curves (Fig. 3F). The dose-response curve for Ad-CA-AMPK-infused rats was flat and shifted downward, and cocaine self-administered rats exhibited a decrease at a threshold dose of 0.1 mg/kg/injection at the peak of the dose-response curve compared with Ad-GFP-infused controls (*p* = 0.002; Fig. 3F). The dose-response curve in Ad-DN-AMPK-infused rats shifted upward, and cocaine self-administering rats exhibited an increase at a threshold dose of 0.03 mg/kg/injection on the ascending limb (*p* = 0.003; Fig. 3F) and a threshold dose of 1 mg/kg/injection on the descending limb of the dose-response curve compared with Ad-GFP-infused controls (*p* = 0.004; Fig. 3F). These data indicated that AMPK activity in the NAcsh regulated cocaine reinforcement under the more demanding FR5 reinforcement schedule and was critical for vertical shifts in the cocaine dose-response curve.

After the dose-response test, cocaine self-administration was re-stabilized at 0.5 mg/kg/injection. Subsequently, the motivation for cocaine was assessed using a progressive-ratio schedule of reinforcement, and the breakpoint was measured at two cocaine injection doses (0.25 and 0.75 mg/kg/injection). The rats generally worked harder for the higher injection dose (dose effect: *F*_1,27_ = 86.920, *p* = 0.000; Fig. 3G), indicating greater motivation to self-administer the higher dose of cocaine. The repeated-measures ANOVA revealed a significant effect of group (*F*_2,27_ = 9.500, *p* = 0.001) and a group × dose interaction (*F*_2,27_ = 8.296, *p* = 0.002). Rats that received intra-NAcsh Ad-DN-AMPK infusions achieved higher breakpoints at the 0.75 mg/kg cocaine injection dose compared with Ad-GFP-infused controls (*p* = 0.028; Fig. 3G), indicating greater motivation for cocaine. Conversely, rats that received intra-NAcsh Ad-CA-AMPK infusions achieved lower breakpoints at both injection doses but exhibited no significant difference from Ad-GFP-infused controls.

### CRTC1 mediates the effects of AMPK on cocaine reinforcement and motivation

**Fig. 4:**
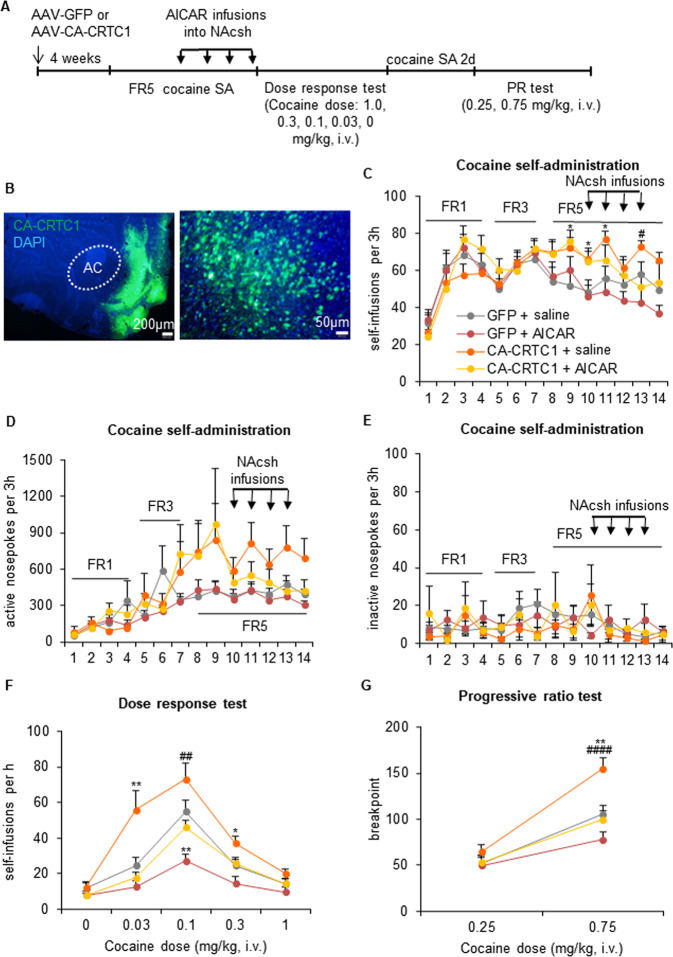
Expression of constitutively active CRTC1 in the NAcsh increases cocaine reinforcement and motivation and rescues the actions of AMPK activation. **A** Timeline of the experiment. Four weeks after the injection of AAV-GFP or AAV-CA-CRTC1 in the NAcsh, the rats were trained to self-administer intravenous injections of cocaine under an FR1 schedule in daily 3 h sessions. The response requirement was gradually increased to FR5, and training continued until cocaine intake stabilized. Following acquisition and stabilization, the rats received intra-NAcsh infusions of saline or the AMPK activator AICAR. The rats then underwent a between-session dose-response test and progressive-ratio test. **B** Photomicrographs of AAV-CA-CRTC1 injection sites in the NAcsh (scale bar = 200 μm [left], 50 μm [right]). Infusions (**C**), active nosepokes (**D**), and inactive nosepokes (**E**) during daily 3 h self-administration sessions. **F** The expression of CA-CRTC1 shifted dose-response curves upward, which was prevented by AICAR treatment (*n* = 8–10/group). **G** The expression of CA-CRTC1 increased breakpoints during the progressive-ratio test, which was prevented by AICAR treatment (*n* = 8–10/group). The data are expressed as mean ± SEM. *Post hoc* analyses were performed using the Tukey test. *p < 0.05, **p < 0.01, compared with GFP + saline group; ^#^*p* < 0.05, ^##^*p* < 0.01, ^####^*p* < 0.0001, compared with CA-CRTC1 + AICAR group.

The above results demonstrated that cocaine reinforcement behaviors resulted in a decrease in AMPK activity, and the suppression of AMPK activity further enhanced self-administration behaviors. Previous studies have revealed that activating AMPK decreased CRTC1 activity in *Caenorhabditis elegans* [15, 34], indicating a negative association between the AMPK and CRTC1. Next, we investigated whether AMPK regulated cocaine reinforcement through the CRTC1 pathway. We bilaterally injected AAV vectors that expressed CA-CRTC1 or GFP control in the NAcsh (Fig. 4A, B). Four weeks later, the rats were trained to self-administer cocaine (0.5 mg/kg/injection) in daily 3-h sessions until cocaine intake stabilized. After stabilization, the rats received the AMPK activator AICAR (2.5 μg per side) or saline infusions in the NAcsh immediately before four consecutive sessions to augment AMPK activity. A two-way repeated-measures ANOVA, with training session as the within-subjects factor and vector (GFP and CA-CRTC1) and AICAR dose (0 and 2.5 μg per side) as between-subjects factors, was performed to analyze cocaine self-administration behavior. The ANOVA of self-infusions (Fig. 4C) revealed effects of a vector × training session interaction (*F*_13,429_ = 2.731, *p* = 0.001), and an AICAR dose × training session interaction (*F*_13,429_ = 1.823, *p* = 0.038). The statistical analyses also revealed a significant effect of vector (*F*_1,33_ = 4.749, *p* = 0.037) and a vector × training session interaction (*F*_13,429_ = 1.859, *p* = 0.033) for active nosepokes (Fig. 4D), but not for inactive nosepokes (*p* > 0.05; Fig. 4E). The expression of CA-CRTC1 in the NAcsh increased cocaine self-administration behavior.

In the between-session dose-response procedure, all cocaine-treated rats exhibited inverted U-shaped dose-response curves that were dose-dependent (dose effect: *F*_4,132_ = 75.821, *p* = 0.000; Fig. 4F). The repeated-measures ANOVA revealed significant effects of vector (*F*_1,33_ = 9.097, *p* = 0.005) and AICAR (*F*_1,33_ = 16.309, *p* = 0.000), a vector × dose interaction (*F*_4,132_ = 4.938, *p* = 0.001), an AICAR × dose interaction (*F*_4,132_ = 9.421, *p* = 0.000), and a vector × AICAR × dose interaction (*F*_4,132_ = 2.544, *p* = 0.043). The expression of CA-CRTC1 increased cocaine self-administration at threshold doses of 0.03 mg/kg/injection (*p* = 0.002; Fig. 4F) and 0.3 mg/kg/injection (*p* = 0.023; Fig. 4F) and blunted the decrease in cocaine self-administration at a threshold dose of 0.1 mg/kg/injection (*p* = 0.005, compared with CA-CRTC1 + AICAR group; Fig. 4F) that was induced by AMPK activation (*p* = 0.008, compared with GFP + saline group; Fig. 4F).

During the progressive-ratio test, the rats generally worked harder for the higher injection dose (dose effect: *F*_1,33_ = 210.281, *p* = 0.000). The repeated-measures ANOVA of breakpoints revealed significant effects of vector (*F*_1,33_ = 6.674, *p* = 0.014) and AICAR (*F*_1,33_ = 8.182, *p* = 0.007), a vector × dose interaction (*F*_1,33_ = 13.510, *p* = 0.001), and an AICAR × dose interaction (*F*_1,33_ = 20.601, *p* = 0.000). The expression of CA-CRTC1 increased breakpoints (*p* = 0.001; Fig. 4G) and blunted the decrease in breakpoint (*p* = 0.000, compared with CA-CRTC1 + AICAR group; Fig. 4G) that was caused by AMPK activation at the 0.75 mg/kg cocaine injection dose.

**Fig. 5:**
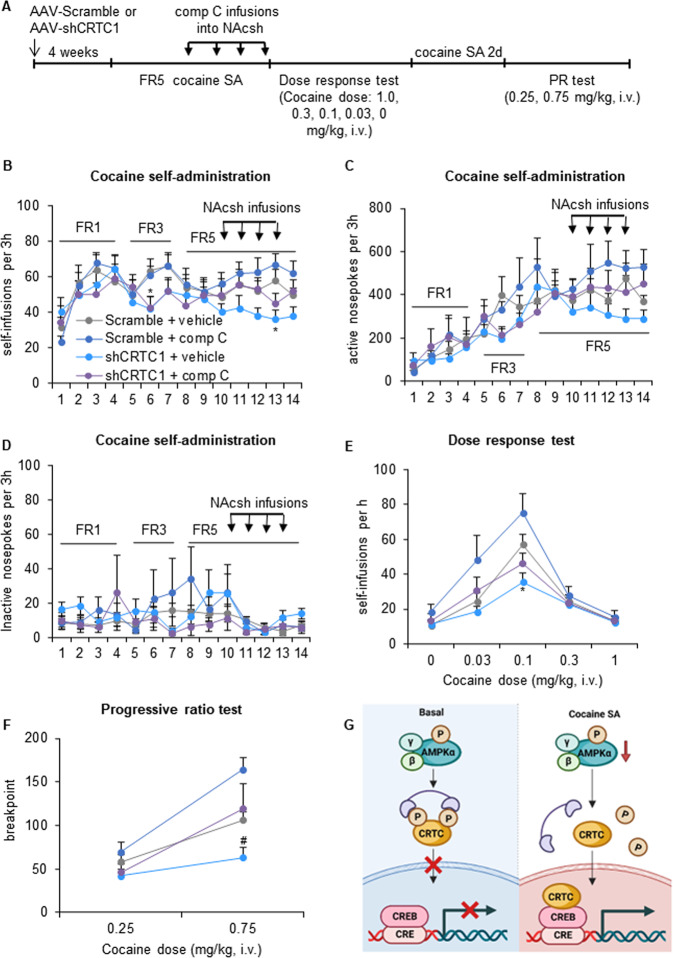
Knockdown of CRTC1 in the NAcsh decreases cocaine reinforcement and motivation and blocks the enhancing effects of AMPK inhibition. **A** Timeline of the experiment. Four weeks after the injection of AAV-Scramble or AAV-shCRTC1 in the NAcsh, the rats were trained to self-administer intravenous injections of cocaine under an FR1 schedule in daily 3 h sessions. The response requirement was gradually increased to FR5, and training continued until cocaine intake stabilized. Following acquisition and stabilization, the rats received intra-NAcsh infusions of vehicle or the AMPK inhibitor compound C. The rats then underwent a between-session dose-response test and progressive-ratio test. Infusions (**B**), active nosepokes (**C**), and inactive nosepokes (**D**) during daily 3 h self-administration sessions. **E** The knockdown of CRTC1 shifted dose-response curves downward, which was rescued by compound C treatment (*n* = 8–12/group). **F** The knockdown of CRTC1 decreased breakpoints during the progressive ratio test, which was prevented by compound C treatment (*n* = 8–12/group). **G** A hypothesized working model of AMPK-CRTC1 signaling in the NAcsh in modulating cocaine reinforcement behaviors. In basal level, phosphorylated CRTC-1 was maintained by high level of AMPK and is sequestered in the cytoplasm. After repeated cocaine self-administration (SA) training, AMPK activity was decreased, promoting the dephosphorylation of CRTC-1. Dephosphorylated CRTC-1 translocates to the nucleus to promote CREB-dependent gene transcription, ultimately contributing to the cocaine reinforcement behaviors. The data are expressed as mean ± SEM. *Post hoc* analyses were performed using the Tukey test. *p < 0.05, compared with Scramble + vehicle group; ^#^*p* < 0.05, compared with shCRTC1 + comp C group.

Another four groups of rats were given bilateral intra-NAcsh infusions of AAV-shCRTC1 or AAV-Scramble 4 weeks before the onset of cocaine self-administration (Fig. 5A). After stabilization, the rats received infusions of the AMPK inhibitor compound C (1.5 μg per side) or vehicle in the NAcsh immediately before four consecutive sessions to attenuate AMPK activity. A two-way repeated-measures ANOVA, with training session as the within-subjects factor and vector (Scramble and shCRTC1) and compound C dose (0 and 1.5 μg per side) as between-subjects factors, was performed to analyze cocaine self-administration behavior. The ANOVA of self-infusions (Fig. 5B) revealed effect of a vector × training session interaction (*F*_13,455_ = 1.869, *p* = 0.032). However, no group differences were found for responding on the active and inactive nosepoke operandum during cocaine self-administration training (*p* > 0.05; Fig. 5C, D). The knockdown of CRTC1 in the NAcsh decreased cocaine self-administration behavior.

In the between-session dose-response procedure, all cocaine-treated rats exhibited inverted U-shaped dose-response curves that were dose-dependent (dose effect: *F*_4,140_ = 84.494, *p* = 0.000; Fig. 5E). The repeated-measures ANOVA revealed significant effects of vector (*F*_1,35_ = 4.178, *p* = 0.049), a vector × dose interaction (*F*_4,140_ = 8.291, *p* = 0.000), and a compound C × dose interaction (*F*_4,140_ = 4.333, *p* = 0.002). The knockdown of CRTC1 decreased cocaine self-administration at a threshold dose of 0.1 mg/kg/injection (*p* = 0.026; Fig. 5E).

During the progressive-ratio test, the rats generally worked harder for the higher injection dose (dose effect: *F*_1,35_ = 86.072, *p* = 0.000; Fig. 5F). The repeated-measures ANOVA of breakpoint revealed significant effects of vector (*F*_1,35_ = 6.315, *p* = 0.017) and compound C (*F*_1,35_ = 6.550, *p* = 0.015), and a compound C × dose interaction (*F*_1,35_ = 14.730, *p* = 0.000). Breakpoints had no change after knockdown of CRTC1, whereas showed a downward trend (*p* = 0.091, compared with Scramble + vehicle group; Fig. 5F). And the knockdown of CRTC1 prevented the increase in breakpoints (*p* = 0.025, compared with shCRTC1 + comp C group; Fig. 5F) that was caused by AMPK inhibition at the 0.75 mg/kg cocaine injection dose.

### Modulation of AMPK activity in the NAcsh has no effect on the motivation for a natural reward

We then investigated whether the regulation of cocaine reinforcement and motivation by AMPK generalizes to natural rewards. Rats were trained to self-administer sucrose solutions on an FR5 schedule and received a similar infusion of AICAR or compound C in the NAcsh. Infusions of AICAR or compound C in the NAcsh had no effect on the rate of sucrose self-administration over the next 4 days (Supplementary Fig. 2A, B). The pharmacological activation or inhibition of AMPK in the NAcsh did not affect the number of sucrose reinforcers earned or breakpoints for sucrose on a progressive-ratio schedule (Supplementary Fig. 2C, D).

## Discussion

The present study revealed a critical role for AMPK in cocaine reinforcement and motivation through the regulation of signaling of the transcriptional coactivator CRTC1. We found that self-administered cocaine decreased AMPK phosphorylation. Augmenting AMPK activity in the NAcsh reduced cocaine self-administration under conditions of higher motivation and led to a downward shift in the dose-response curve and decrease in breakpoint in a progressive-ratio schedule, which were rescued by increasing CRTC1 activity. Decreasing AMPK activity in the NAcsh enhanced cocaine self-administration under conditions of higher motivation and led to an upward shift in the dose-response curve and increase in breakpoint, which were prevented by CRTC1 knockdown. Altogether, our findings reveal a novel role of AMPK in the regulation of cocaine reinforcement behaviors.

Numerous studies revealed the importance of AMPK for the regulation of peripheral glucose metabolism [5, 35]. The activation of AMPK promotes the catabolic process and prevents the anabolic process [35]. Emerging evidence indicates a role for AMPK in the neuroendocrine control of organismal metabolism with aging and food intake [36–38], even in the pathogenesis of several neurodegenerative disorders, such as Alzheimer’s disease and amyotrophic lateral sclerosis [39, 40]. Although rare research has explored the expression pattern of AMPK in the postmortem brain samples from cocaine abusers, there are some clinical studies suggesting that AMPK is involved in substance use disorders. Schmitz et al. [41] divided 18 patients with cocaine use disorder into pioglitazone (an indirect activator of AMPK) group and placebo group. A significant decrease in cocaine craving was found in patients who received pioglitazone, suggesting that AMPK activation prevented the motivation for cocaine use. Similarly, rodent studies showed that the indirect pharmacological stimulation of AMPK reduced the acquisition of cocaine-induced conditioned place preference [42] and cocaine-induced hyperlocomotion [43]. In the present study, we found that cocaine self-administration training reduced p-AMPK levels up to 7 days of withdrawal, suggesting a decrease in AMPK activity and possibly an increase in energy metabolism in the NAcsh during cocaine self-administration training. However, an opposite expression of p-AMPK in the NAc was found 30 min after acute cocaine treatment in vitro and in vivo [44], suggesting that different manipulations may affect the AMPK activity differentially. AICAR and compound C are extensively used as pharmacological agents to modulate AMPK activity, but there are also some studies reporting an AMPK-independent effect of AICAR in the liver [31, 32], and compound C has been shown to inhibit several other kinases such as ERK8 and FGFR1 which are involved in the cocaine actions [33, 45, 46]. Therefore, we further applied Ad-CA-AMPK and Ad-DN-AMPK to directly modulate AMPK activity in the NAcsh to avoid the interference of other factors. Modulating AMPK activity produced a vertical shift in the cocaine self-administration dose-response curve and affected the motivation to obtain cocaine under a progressive-ratio schedule, verifying the necessity of AMPK for cocaine reinforcement behaviors.

Accumulating evidence indicates that AMPK is essential for transcriptional regulation through the phosphorylation of some transcription factors and coactivators, histone deacetylase families, and histones themselves [5]. AMPK networks are composed of different downstream signaling factors that regulate various processes, such as protein metabolism, lipid metabolism, glucose metabolism, and mitochondrial homeostasis [35]. The CRTC family is a direct AMPK target and has emerged as a unique CREB coactivator that binds to the basic leucine zipper region of CREB [17]. Phosphorylated CRTCs are sequestered in the cytoplasm, whereas dephosphorylated CRTCs translocate to the nucleus, promoting CREB-dependent gene transcription [47]. Three CRTCs have been identified in humans, among which CRTC1 is an isoform that is most abundantly expressed in the central nervous system [17, 48]. Diverse functions are regulated by CTRC1 signaling, including mitochondrial metabolism and longevity [15, 34], contextual long-term memory formation [49], and the strengthening of new memories [50]. The AMPK/CRTC1 pathway was shown to play a role in modulating longevity in *Caenorhabditis elegans* [15, 34]. However, little is known about the function of this pathway in mammals. In our research, CRTC1 activation led to an upward shift in the dose-response curve and increase in breakpoint in a progressive-ratio schedule, which was prevented by augmenting AMPK. The decrease in cocaine reinforcement behaviors that was caused by CRTC1 inhibition was also rescued by inhibiting AMPK. Few studies have investigated the role of AMPK/CRTC-1 signaling in the reward system. Activated AMPK could inhibit dopamine release in dopaminergic neurons [51], and CRTCs shows a positive association with the dopamine effect in renal proximal tubule [52]. We speculate that during cocaine self-administration training, dephosphorylated CRTC1 that is caused by a decrease in p-AMPK promotes dopamine release in the NAcsh. In turn, the augmentation of AMPK possibly inhibits dopamine release by blocking CRTC1 from entering the nucleus, resulting in a reduction of cocaine reinforcement behaviors (Fig. 5G). Future work is worthy to further investigate the distribution of AMPK in subpopulations of cells in the NAcsh, and to study the specific influence of AMPK in distinct cells on the cocaine-related behaviors. In addition, it should be noted that only male rats were used in the present study, and whether there would be sex differences in the effect of AMPK-CRTC1 signaling in the NAcsh on the motivation for cocaine needs more investigations in the future.

AMPK in different brain regions may be involved in different processes of substance use disorders. Our previous study showed that cue-induced reinstatement of cocaine seeking increased p-AMPK levels in the NAcc rather than NAcsh. Stimulating AMPK activity in the NAcc inhibited cue-induced reinstatement of cocaine seeking, whereas no effect was found after inhibiting AMPK in the NAcsh [13]. The present study demonstrated that AMPK in the NAcsh mediated the cocaine reinforcement behaviors. The heterogeneity of findings may be attributable to different projections between the NAcc and NAcsh [53, 54]. The NAcc is involved in the evaluation of reward and driving reward-related motor actions, mainly receiving glutamatergic inputs from dorsal prefrontal cortex and basolateral amygdala, γ-aminobutyric acidergic (GABAergic) inputs from ventral pallidum, and dopaminergic afferents from ventral tegmental area (VTA) [55]. Numerous studies have identified the role of NAcc-composed circuits in cue-induced reinstatement of drug seeking [1]. Afferent from the prelimbic cortex promoted the synaptic strength of NAcc neurons through recruiting of Ca^2+^-impermeable AMPA receptors during prolonged abstinence from cocaine, and optogenetic inhibition of this circuit reduced cue-induced cocaine seeking at late abstinence time [56]. Different from the NAcc, the NAcsh plays an important role in motivation and reward-related process [55]. Various afferents and efferents are found in the NAcsh, such as inputs from the ventromedial prefrontal cortex, ventral hippocampus, and the VTA, and outputs to the hypothalamus. A recent study showed the engagement of GABAergic projections from the VTA to the ventral NAcsh (vNAcsh) in natural reward reinforcement behaviors [57]. Optogenetic activation of VTA GABAergic projections to the vNAcsh increased reward reinforcing behaviors, while no effect was found after photomanipulation of VTA GABAergic inputs to the NAcc. Furthermore, this circuit activity during reinforcement had an inverse relationship with cholinergic interneurons (CINs) in the NAcsh and acetylcholine (ACh) release, indicating that VTA GABAergic projection to the vNAcsh promoted the reinforcing behaviors possibly through inhibiting the CINs and ACh release. Although it is not clear whether VTA to NAcsh circuit has the same effect in cocaine reinforcement, it provides some insight about how AMPK would be involved in the NAcsh-composed circuits during cocaine reinforcement behaviors. Previous studies reported that AMPK can contribute to neuronal excitability through direct phosphorylation and regulation of GABA-A receptors [58]. It would be worthy to further investigate whether AMPK signaling mediates the cocaine reinforcement behaviors through modulating the activity of distinct afferents to NAcsh.

Altogether, our results indicate that AMPK-CRTC1 signaling regulates cocaine reinforcement and motivation. These findings provide further insights into the molecular mechanisms that underlie the development and persistence of cocaine reinforcement behaviors, implicate AMPK as a potential therapeutic target, and have promising implications for the treatment of cocaine use disorders.

## Supplementary information

Supplementary Information
