# Supplementary material for: Novel role of AMPK in cocaine reinforcement via regulating CRTC1
Source: Transl Psychiatry. 2022 Dec 31;12:530. doi: 10.1038/s41398-022-02299-w (PMC9805446; doi:10.1038/s41398-022-02299-w)
Supplement: Supplementary file 1 — Supplementary Information [file 41398_2022_2299_MOESM1_ESM.docx]

**Supplementary Information**

**Novel role of AMPK in cocaine reinforcement via regulating CRTC1**

**Xiao-Xing Liu, Ph.D.,^1^ Fang-Lin Liu, B.S.,^2^ Xin Li, B.S.,^2^ Tang-Sheng Lu, M.D.,^2^** **Yi-Xiao Luo, Ph.D.,^2, 3^ Min Jian, Ph.D.,^2^ Kai Yuan, Ph.D.,^1^ Shi-Qiu Meng, Ph.D.,^2^ Yan-Ping Bao, Ph.D.,^2, 4^ Jie Shi, Ph.D.,^2^ Lin Lu, M.D., Ph.D.,^1, 5^ Ying Han, Ph.D.,^2,^ ***

**This file includes:**

**Supplementary Figures and Figure Legends**

**Supplementary Figures and Figure Legends**

**
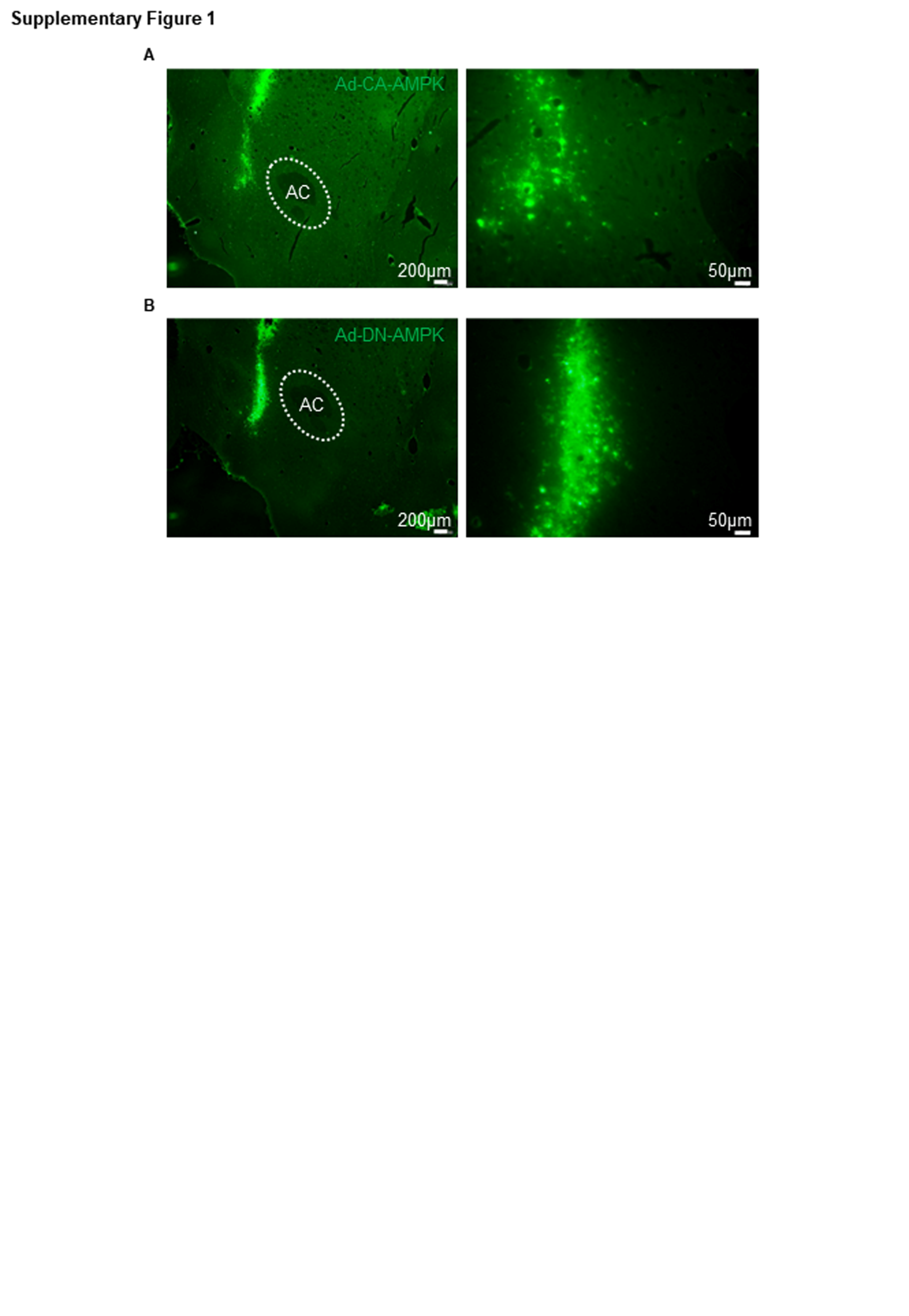
**

**Supplementary Figure 1. Representative photographs of the injection sites in the NAcsh.** These figures show representative micrographs of Ad-CA-AMPK **(A)** and Ad-DN-AMPK **(B)** microinjections in the nucleus accumbens shell (NAcsh). Scale bar = 200 μm (left), 50 μm (right).

**
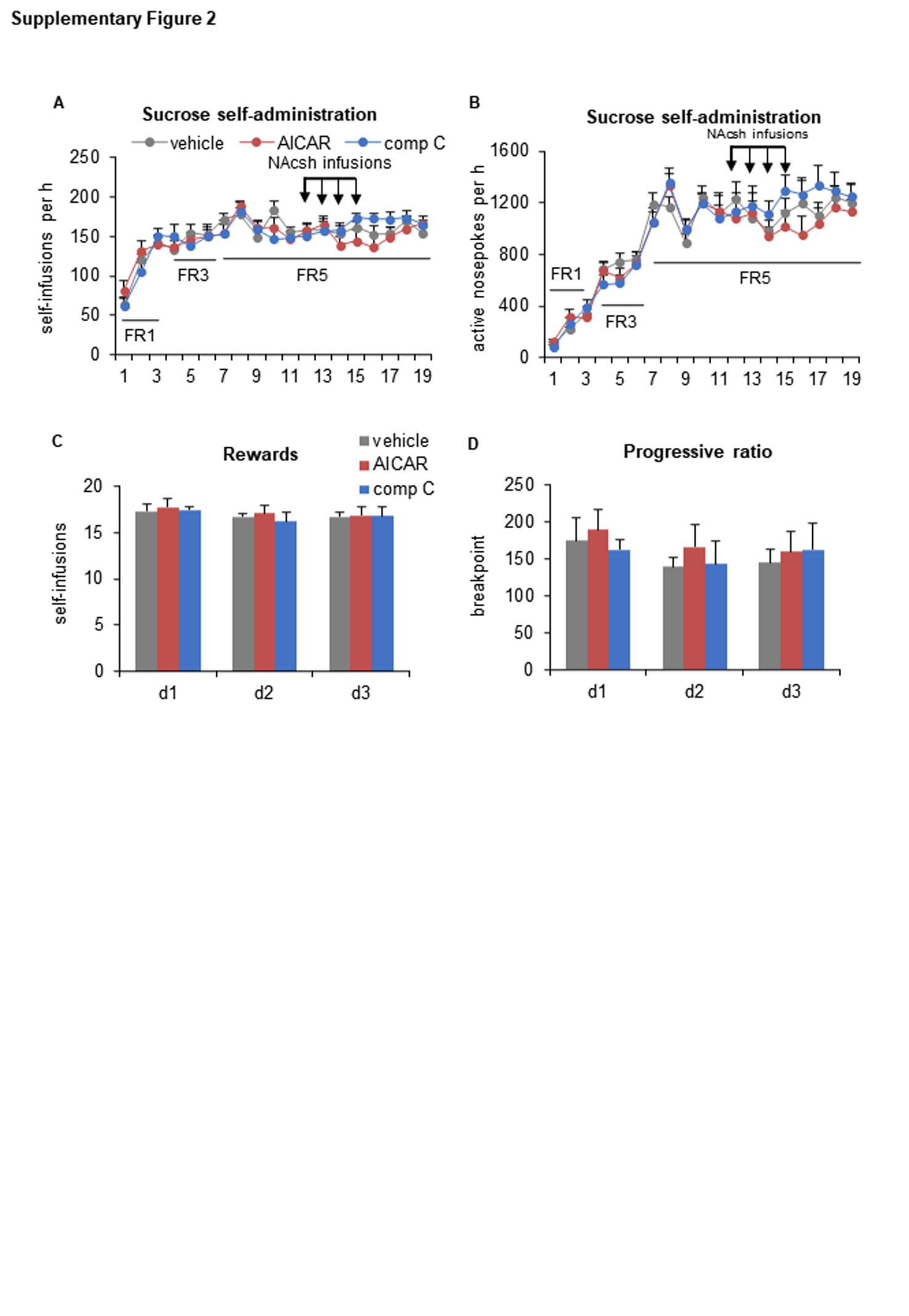
**

**Supplementary Figure 2. Modulation of AMPK activity in the NAcsh fails to alter motivation for natural reward.** **A and B**, Repeated infusions of AICAR (2.5 μg/side) or compound C (1.5 μg/side) in the NAcsh had no effect on sucrose self-administration rates on an FR5 schedule after the cessation of treatment (*n* = 9-10/group). **C**, Repeated intra-NAcsh infusions of AICAR or compound C had no effect on the number of sucrose rewards earned in over 2 h of operant testing. **D**, Repeated intra-NAcsh infusions of AICAR or compound C had no effect on progressive-ratio responding for sucrose in over 2 h of operant testing. The data are expressed as mean ± SEM.
